# Entrainment of local synchrony reveals a causal role for high-beta right frontal oscillations in human visual consciousness

**DOI:** 10.1038/s41598-019-49673-1

**Published:** 2019-10-10

**Authors:** Marine Vernet, Chloé Stengel, Romain Quentin, Julià L. Amengual, Antoni Valero-Cabré

**Affiliations:** 1 Institut du Cerveau et de la Moelle Epinière (ICM), CNRS UMR 7225, INSERM U 1127 and Sorbonne Université, Paris, France; 2 Institut des Sciences Cognitives Marc Jeannerod, CNRS UMR 5229 and Université Claude Bernard, Lyon, France; 3 Laboratory for Cerebral Dynamics Plasticity and Rehabilitation, Boston University, School of Medicine, Boston, MA USA; 4 Cognitive Neuroscience and Information Technology Research Program, Open University of Catalonia (UOC), Barcelona, Spain

**Keywords:** Consciousness, Perception

## Abstract

Prior evidence supports a critical role of oscillatory activity in visual cognition, but are cerebral oscillations simply correlated or causally linked to our ability to consciously acknowledge the presence of a target in our visual field? Here, EEG signals were recorded on humans performing a visual detection task, while they received brief patterns of *rhythmic* or *random* transcranial magnetic stimulation (TMS) delivered to the right Frontal Eye Field (FEF) prior to the onset of a lateralized target. TMS entrained oscillations, i.e., increased high-beta power and phase alignment (the latter to a higher extent for *rhythmic* high-beta patterns than *random* patterns) while also boosting visual detection sensitivity. Considering post-hoc only those participants in which rhythmic stimulation enhanced visual detection, the magnitude of high-beta entrainment correlated with left visual performance increases. Our study provides evidence in favor of a causal link between high-beta oscillatory activity in the Frontal Eye Field and visual detection. Furthermore, it supports future applications of brain stimulation to manipulate local synchrony and improve or restore impaired visual behaviors.

## Introduction

Within the last decade, there has been increasing interest in the involvement of oscillations and synchronization in information coding^1,2^. In the research field of attention and perception, occipital alpha^3–5^, gamma^3,5^ and theta^6^ oscillations, and also fronto-parietal beta and gamma oscillatory modes^7,8^, coordinated by theta oscillations^9^ have been associated with the ability to consciously acknowledge the presence of a visual target. Additionally, local and interregional oscillations at these frequency bands are thought to code for specific cognitive processes. For example, in monkeys, episodes of fronto-parietal synchronization at 30 and 50 Hz have been correlated with top-down and bottom-up attention orientation, during a visual search task and a pop-out visual task, respectively^7^. Similarly in humans, 30-Hz rhythmic patterns of Transcranial Magnetic Stimulation (TMS) delivered to a right frontal region increased conscious visual sensitivity, suggesting that high-beta synchrony^10^ within a fronto-parietal network^11,12^ is causally involved in conscious visual perception. However, are these behavioral effects causally mediated by the engagement of frequency-specific oscillatory activity? Or, do cortical oscillations simply represent an epiphenomenon devoid of any direct implication concerning visual cognition and associated visually-guided behaviors^13^?

To address the question of the causal role of local oscillations in conscious perception, we designed an experiment in which participants performed a visual detection task while receiving rhythmic transcranial magnetic stimulation (rhythmic TMS)^14,15^. This noninvasive perturbation approach allows testing relevant causal contributions of rhythmic activity at specific time-windows in circumscribed brain regions during human cognitive processing. In our experiment, rhythmic TMS patterns were designed to entrain short episodes of high-beta rhythmic activity (30 Hz) in the right Frontal Eye Field (FEF), a key node of the dorsal attentional network^16^ involved in the modulation of visual perception in monkeys^17^ and humans^18–20^. Combined TMS-EEG recordings have shown the entrainment of short-lasting alpha oscillations (~10 Hz) by noninvasive rhythmic stimulation delivered at alpha frequency to posterior parietal locations at rest, i.e., in participants not engaged in any specific task-driven behavior^21^. Beta frequencies in motor or memory systems have been successfully entrained in recent studies^22,23^. However, entrainment at such frequencies over regions specifically relevant for visual cognition and conscious access remains to be explored.

In the present study, a group of right-handed healthy human participants performed a visual detection task. Before the onset of the visual target, either active or sham TMS bursts were applied over the right FEF. We compared the neurophysiological (*EEG recordings*) and behavioral (*visual detection sensitivity*) effects of *rhythmic* stimulation patterns, composed of 4 TMS pulses at 30 Hz, to the effects of *random* TMS patterns (with identical time onset, duration, and pulse number, but providing arrhythmic activity). We hypothesized that rhythmic right frontal TMS patterns would entrain oscillations by progressively aligning the phases of local oscillators at the frequency of the stimulation source^21,24^. Such effect would be captured by increases of high-beta power, inter-trial coherence, and amplitude of evoked oscillations (see methods section for details), which should be larger for rhythmic than for random TMS patterns. Furthermore, on the basis of a rich literature showing that the effects of frontal lesions or TMS perturbations on attention and perception are often lateralized (for a review see^25^), we predicted improvements of visual performance under the impact of high-beta rhythmic patterns, restricted to the contralateral left visual hemifield. Finally, we predicted an association between visual detection improvement and the amplitude of evoked high-beta oscillations measured by the end of the stimulation burst, when entrainment has been previously shown to reach its maximum^21^. Together, these results would provide evidence in favor of a causal contribution of oscillatory activity to specific aspects of visual cognition. It would also support future manipulations of local synchrony to improve or restore specific aspects of perceptual performance, encoded by oscillation-based mechanisms.

## Materials and Methods

### Participants

Fourteen right-handed participants (9 women and 5 men) between 20 and 34 years old (24 ± 4) took part in the study. Between ten and fourteen participants have been enrolled in previous studies demonstrating the effects of rhythmic stimulation on cortical oscillations^21^ or on visual perception^10^, motivating the number of participants included in the present study. Participants underwent all experimental conditions (within-subject experimental design), were naïve to the purpose of the experiment, reported no history of neurological or psychiatric disorders, had normal or corrected-to-normal vision, and participated voluntarily after providing written informed consent. The protocol (C08-45) was reviewed by the Inserm (Institut National de la Santé et la Recherche Scientifique) ethical committee and was approved by an Institutional Review Board (CPP Ile de France 1) in accordance with the Declaration of Helsinki.

### Apparatus

Participants were seated with their head positioned on a chin-rest and their eyes directed towards a computer screen (22″) 57 cm away. A custom-made script, using the MATLAB (Mathworks) Psychtoolbox^26^, ran on a desktop computer (HP Z800, Hewlett Packard). It synchronized the presentation of visual stimuli on the computer screen, the pulses delivered by two biphasic repetitive TMS devices (SuperRapid, Magstim) attached to standard 70 mm figure-of-eight coils operated via a trigger-synchronization device (Master 8, A.M.P.I.), a remote gaze tracking capture system (Eyelink 1000, SR Research), and EEG recordings performed with TMS-compatible equipment (BrainAmp DC, BrainVision Recording Software, EasyCap and Ag/AgCl sintered ring electrodes, BrainProducts GmbH). Additionally, a frameless neuronavigation system (Brainsight, Rogue Research) was used throughout the experiment to deliver TMS on precise standardized coordinates corresponding to the right FEF.

### Visual detection paradigm

A visual detection paradigm similar to the one employed in prior studies was used^10–12,18,27^. Each session included a titration block, a training block, and 4 experimental blocks. The latter blocks assessed the effects of rhythmic/random TMS on EEG signals and visual detection (2 blocks for each stimulation pattern). Each block was divided into sub-blocks of 20 trials. Calibration and training blocks were tailored in length to each participant and completed once stable performance was reached. Experimental blocks included a fixed number of 140 trials divided in 7 sub-blocks.

**Figure 1:**
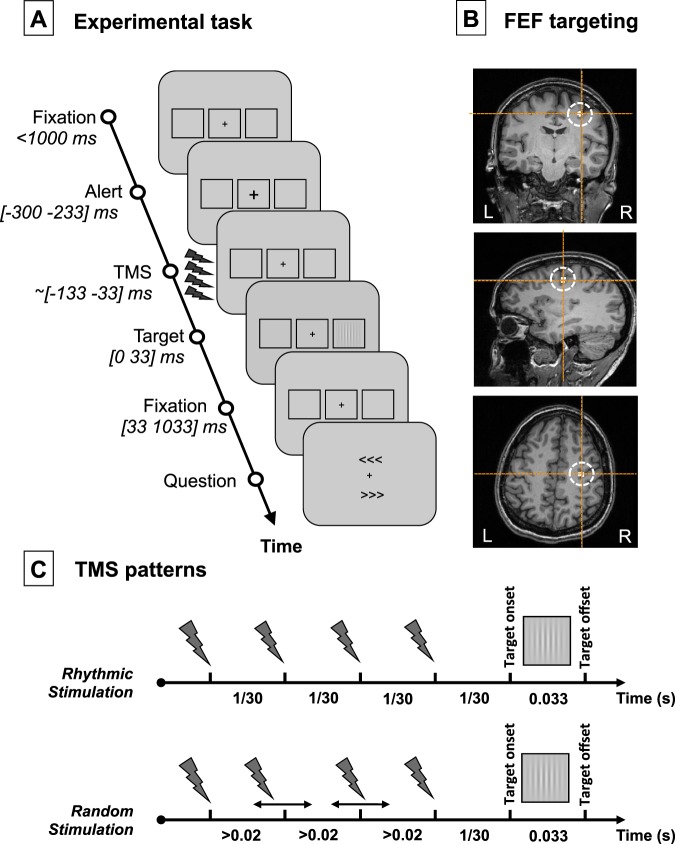
Experimental task, TMS targeted cortical region and stimulation patterns. (**A**) Visual detection task performed by participants. After a period of fixation, a central cross became slightly larger (alert cue) to alert participants of an upcoming event. Then active/sham rhythmic/random TMS patterns were delivered to the right FEF region prior to the presentation of a visual target at the center of a right/left placeholder. Participants were requested to indicate whether they did or did not perceive a target and, if they did, where it appeared (no target perceived/target perceived on the right/target perceived on the left). Notice that in 20% of the trials (“catch trials”), no target was presented in any of the two placeholders. (**B**) Coronal, sagittal and axial T1-3D MRI sections from a representative participant generated by the frameless stereotaxic neuronavigation system showing the localization of the right FEF, stimulated in our experiment (Talairach coordinates X = 31, Y = −2, Z = 47^29^). (**C**) Schematic representation of the temporal distribution of the 4-pulse bursts employed for the 30 Hz *rhythmic* and the *random* stimulation conditions. Contrasting the behavioral (visual detection sensitivity) and electrophysiological (EEG) impact elicited by these two patterns isolates the effects of 30 Hz FEF activity (only present in rhythmic bursts) from those induced by 4 TMS pulses delivered during a 100 ms interval (featured by both rhythmic and random bursts).

Each trial (see Fig. 1A) started with a gray resting screen (luminance: 31 cd/m2, 2500 ms) followed by a fixation screen (randomly lasting between 1000 and 1500 ms). A fixation cross (0.5 × 0.5°) was displayed at the center of the screen along with two lateral placeholders (6.0 × 5.5°, eccentricity 8.5°). The fixation cross became slightly larger (0.7 × 0.7°, 66 ms) to alert participants of an upcoming event. Then, following a fixed inter-stimulus interval (233 ms), a target could appear for a brief period of time (33 ms) in the center of one of the two placeholders (40% left trials, 40% right trials, 20% no target or “catch” trials). The target was a low-contrast Gabor stimulus made of vertical lines (0.5°/cycle sinusoidal spatial frequency, 0.6° exponential standard deviation, minimum and maximum Michelson contrast of 0.005 and 1, respectively).

As in prior studies^10,11,18^, participants were asked to report whether they perceived the target or not and, if they did, where on the screen it appeared (detection task). To do so, two arrow-like signs (“<<<” and “>>>”), pointing to the left and to the right, were simultaneously presented below and above the fixation cross. Participants used 3 keys of a keyboard to answer: an upper key “d”, a lower key “c” and the space bar, which they operated with the middle, index, and thumb fingers of their left hand, respectively. Participants were requested to respond either by pressing the space bar if they did not see the stimulus, or by pressing “d”/“c” to select the upper/lower arrow-like sign pointing to the placeholder where they had perceived the target. The location of each arrow, above or below the fixation point, was randomized across trials. The response of the participant ended the trial.

Based on previously used procedures^10,28^, a titration block served to estimate the contrast for each participant where ~50% of the visual targets were detected and reported (visual detection threshold). This procedure was performed under the effect of sham TMS patterns delivered before the target onset to the right FEF, identical to those used during the experiment (see full details on the TMS procedure section below). Participants initiated the titration with a high contrast target. A one-up/one-down staircase procedure was employed to adjust stimulus contrast in search of the threshold. The initial contrast step was equal to the initial contrast level. Then, contrast steps were divided by two on each reversal, but were always kept higher than 0.005 Michelson contrast units. We considered the 50% threshold to be reached when the last five consecutively tested contrasts were not different by more than 0.01 Michelson contrast units. This procedure was repeated twice. If the difference between the two estimated thresholds was lower than 0.01 Michelson units, the calibration procedure was terminated at the end of the ongoing sub-block and the average of the two last thresholds was taken as the final 50% threshold. If the difference between the two measures proved equal to or higher than 0.01 Michelson contrast units, the threshold was determined again. A short break was allowed at the end of each sub-block of testing.

During the ensuing training block, active or sham TMS was delivered on the right FEF (see further details on the TMS procedure section below). Half of the trials for each condition (left target, right target, no target) were performed under the effects of active TMS, whereas the other half were performed under the impact of sham TMS. Within each block, all types of trials, i.e., three visual targets (left, right, no target) performed under two TMS conditions (real, sham), were carried out in randomized order. This training block aimed to further familiarize the participant with the stimulation and to check the consistency of visual detection performance of sham trials (previously titrated at a 50% correct detection performance under sham TMS) when both active and sham TMS trials were randomly mixed-up during the same block. At the end of each sub-block, participants were allowed to take a rest and the experimenter re-adjusted target contrast if necessary.

Once participants carried out the training task consistently and according to the established titration level, they were invited to perform 4 experimental blocks of 140 trials (each organized in 7 sub-blocks of 20 trials). These were identical to the training block, except that the target contrast was kept constant and short breaks were allowed every two sub-blocks.

For fixation control purposes, eye movements were monitored. Fixation was considered broken when participants’ gaze was recorded outside a 2° radius of the fixation cross any time between the alerting cue onset and the target offset. When this occurred, participants received an alert message; that specific trial was randomized with the rest of the trials left in the sub-block and repeated. At each break, participants received an alert signal if their false alarm rate (i.e., reporting having seen a target when no target was presented) was higher than 50% (only during the calibration and training blocks), and they were also informed on the percentage of target location errors and the percentage of incorrect gaze central fixations (at the end of each block and throughout the experiment).

### Noninvasive brain stimulation procedures with rhythmic TMS

Active TMS bursts were delivered to the right FEF. The right FEF region (see Fig. 1B) was localized on each individual T1-weighted MRI scan (3T Siemens MPRAGE, flip angle = 9, TR = 2300 ms, TE = 4.18 ms, slice thickness = 1 mm, isovoxel) using averaged Talairach coordinates x = 31, y = −2, z = 47^29^ and a 0.5 cm radius spherical Region of Interest (ROI). At all times, the TMS coil was held tangentially to the skull, with a rostral-to-caudal and lateral-to-medial orientation at ~45° with respect to the longitudinal interhemispheric fissure (i.e., ~parallel to the central sulcus), and kept within an area of ~1–2 mm radius from the right FEF by means of a frameless neuronavigation system.

Sham TMS bursts were randomly interleaved during the same block. They were delivered by a second TMS coil, placed next to the right FEF site and oriented perpendicular to the scalp, which prevented the magnetic field from reaching the skull and stimulating the brain. Acoustic stimulation related to the coil discharge noise was diminished by having participants wear earplugs; skull bone vibration when the coil is discharging could also potentially contribute to auditory evoked potentials and entrainment (for a review, see^30^, see also^31^). Nevertheless, those two concerns were limited by having the sham coil also in contact with the skull, thus mimicking the accompanying auditory and somatosensory effects of active TMS, even if some slight differences remain^32^. Furthermore, to minimize attentiveness to the TMS itself, participants were familiarized during the training with the sensations associated to transcranial stimulation. It should be also noted that during the experiment, participants were largely attentive to the challenging conscious visual detection task they were required to perform.

Stimulation consisted of either rhythmic or random bursts made of 4 consecutive TMS pulses with a total duration of 100 ms. The last pulse was delivered 1/30th of a second (i.e., a cycle of a 30 Hz oscillation) prior to the onset of the visual target. Rhythmic patterns consisted of four pulses uniformly distributed to produce a regular 30 Hz burst. Random patterns, which were used as control patterns to isolate the specific contribution of stimulation frequency, had their 1^st^ and 4^th^ pulses delivered at the same timing as in the rhythmic patterns. In contrast, the 2^nd^ and 3^rd^ pulses of the burst were randomly delivered in the interval left between the 1^st^ and 4^th^ pulses respecting the following constraints: (1) a minimum interval of 20 milliseconds between two contiguous pulses, to ensure the effective recharge of TMS machines capacitors; and (2) a minimum interval of three milliseconds between the timing of each of the two middle pulses and the timing that would have been held in pure 30 Hz rhythmic patterns (see Fig. 1C). Rhythmic and random TMS were delivered in distinct experimental blocks performed in a counterbalanced order (7 participants started with rhythmic TMS block and 7 with random TMS block).

Our TMS design (within-block active/sham conditions; between-block rhythmic/random conditions) has been successfully tested in several TMS studies combining rhythmic stimulation and perceptual behaviors^10–12,27^ in absence of EEG recordings. The type of stimulation delivered on each trial (*active* vs. *sham*) was randomized online by the computer in control of the behavioral paradigm. This within-block active/sham design controlled for natural fluctuations of sustained attention and arousal level across the session.This provided an opportunity to subtract the potential impact caused by the unspecific effects associated to TMS delivery. This also precluded any possibility for neither participants nor the TMS operator to anticipate the type of TMS (*active* vs. *sham*) delivered on a given trial, hence protecting the experiment from conscious/unconscious biases. Interestingly, our preliminary testing revealed that when tested in separate blocks, differences between *rhythmic* vs. *random* TMS patterns passed unnoticed to participants. Indeed, debriefing performed in past reports^10–12,27^ and also in the current study confirmed that participants (whose attention during the session was captured by a highly demanding near-threshold visual detection task) were unaware of slight temporal differences between the delivered stimulation patterns (rhythmic vs. random) tested in separated blocks and presented in counterbalanced order.

Stimulation intensity was set up at a fixed value of 55% of the maximal stimulator output (MSO), instead of being adjusted to the individual resting motor threshold (RMT). Scalp-to-cortex distance is known to account for variability of the motor threshold^33^; nevertheless, other factors are probably at play for determining the excitability of other brain areas. Indeed, TMS-measured excitability in M1 predicts poorly the excitability of other areas^34,35^. Our previous studies demonstrated behavioral effects at the group level using a fixed intensity of 45% MSO^10,11^. In the present study, the intensity of 55% took into account the estimated increased coil-to-cortex distance due to the presence of the EEG cap. However, to allow comparison with other studies^36^, the resting motor threshold (RMT) for the left *abductor pollicis brevis* (APB) muscle was determined on each participant at the end of the experiment as the minimum intensity at which TMS pulses applied on the right primary motor cortex (M1) yielded an activation of the APB in at least 50% of the attempts (RMT = 72 ± 9% MSO). The stimulation intensity applied to the right FEF and employed in the experiment corresponded on average to 78 ± 12% of each participant’s motor threshold. Post-hoc analyses confirmed that the magnitude of the behavioral and physiological effects reported in the present study was not significantly correlated with individual RMT (p  = 0.69).

### EEG recordings and analyses

#### EEG recordings

EEG activity was continuously acquired from 60 scalp electrodes with the reference placed on the tip of the nose and the ground located on the left earlobe. Electrooculogram (EOG) was recorded with 4 additional electrodes (on the right and left temples and above and below one eye). Skin/electrode impedance was maintained below 5 kOhm. The signal was digitized at a sampling rate of 5000 Hz.

#### EEG preprocessing and artifact removal

EEG signals were analyzed with MATLAB (R2013a), EEGLAB (v10.2.5.5.b)^37^ and FieldTrip^38^ according to the following procedure. First, EEG data were epoched around the onset of the visual target ([−2 s, +2 s]). Then a 2^nd^ order infinite impulse response (IIR) Butterworth filter (pass-band between 1 and 50 Hz) was used with a forward-backward filtering to maintain a zero phase shift. This filter was not applied to time windows containing TMS pulses ([−4 ms, +12 ms] centered at each of the four pulses’ onset). Afterwards, TMS electromagnetic artifacts were eliminated from the EEG signals by removing this time window and performing a linear interpolation^30,39,40^. The length of this interval (16 ms for each of the four pulses) was chosen after examination of the raw data in every participant. Data were then resampled at 500 Hz. Trials contaminated by blinks or muscle artifacts were identified by visual inspection, and removed. For each experimental condition, residual artifacts were segregated from physiological responses using a common Independent Component Analysis (ICA). For each participant, 8 ± 4 components (range from 3 to 16) related to electrical artifacts were identified by activity strongly peaking in the vicinity of the stimulation site shortly after each TMS pulse, and by a spectrum covering a restricted frequency range with strong harmonics^41,42^. Once these components were removed, cleaned EEG signals were calculated back at the electrode level. This procedure of TMS artifacts removal was applied to all EEG trials, whether the magnetic stimulation applied was active or sham.

#### Synchrony entrainment: modulations of power and inter-trial coherence

Once data cleaning was completed, two types of procedures were performed for each analysis: the first one included data from all 60 EEG scalp electrodes, whereas the second one concerned only EEG signals from the closest electrode to the stimulated right FEF area (i.e., electrode FC2).

**Figure 2:**
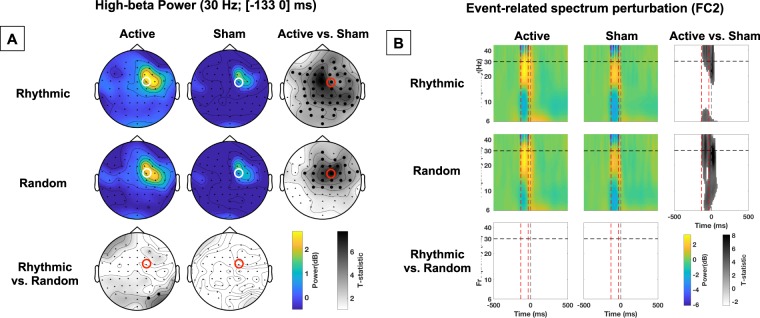
Impact of active vs. sham, rhythmic vs. random, right FEF stimulation on power. (**A**) Topographical maps representing power at 30 Hz during the [−133 0] ms pre-target onset time-window. The location of electrode FC2 (closest to the stimulated right FEF site), is indicated with a red or white open circle. On the statistical maps, electrodes from the topographic views for which EEG signals proved significantly different between conditions are signaled with bold black dots. Notice the increase of 30 Hz power for active vs. sham stimulation. **(B**) Event-related spectrum perturbation (ERSP) calculated for scalp electrode FC2. Red dashed vertical lines signal the onset and offset of the 4-pulse stimulation burst; the grey dashed vertical line indicates the visual target onset. The horizontal black dashed line signals the frequency of 30 Hz at which active rhythmic patterns were delivered. On the statistical maps, grey colors indicate statistically significant difference between conditions. Notice that both active rhythmic and active random stimulation increased power at ~30 Hz during stimulation.

**Figure 3:**
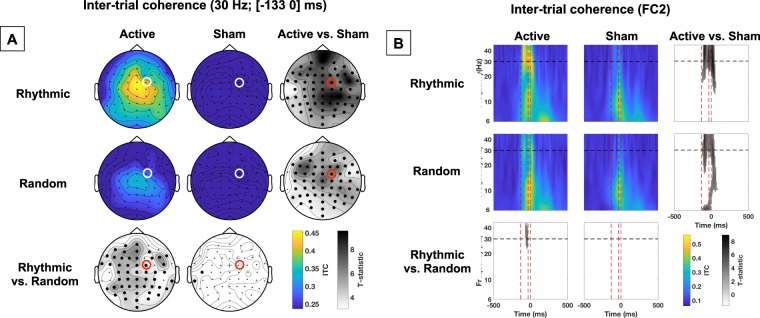
Impact of active vs. sham, rhythmic vs. random, right FEF stimulation on phase alignment (inter-trial coherence). **(A)** Topographical maps representing inter-trial coherence (ITC) of 30 Hz EEG activity during the [−133 0] ms pre-target onset time window. The location of electrode FC2 (closest to the stimulated right FEF site), is indicated with a red or white open circle. On the statistical maps, electrodes from the topographic views for which EEG signals proved significantly different between conditions are signaled with bold black dots; cross signs indicate marginally significant differences. **(B)** Inter-trial coherence (ITC) at scalp electrode FC2 throughout frequency bands and time windows. Red dashed vertical lines signal the onset and offset of the 4-pulse stimulation burst; the grey dashed vertical line indicate the visual target onset. The horizontal black dashed line signals the frequency of 30 Hz at which active rhythmic patterns were delivered. On the statistical maps, grey colors indicate statistically significant difference between conditions. Notice that a direct comparison between active *rhythmic* and *random* stimulation shows higher ITC for active *rhythmic* stimulation.

The procedure to evaluate power (estimating the amount of rhythmic activity) and inter-trial coherence (evaluating the phase alignment of rhythmic activity) started with a time-frequency EEG analysis based on pure 3-cycles Morlet wavelets during a [−500 500] ms time interval and within a [6 50] Hz frequency window. The EEG baseline for the calculation of power was defined as the activity preceding the onset of the alerting central cue within the [−500 −300] ms time window (see Fig. 1 for details on the timing of events). We performed two types of analyses: the first one concerned the frequency (30 Hz) and time of interest (beginning of burst delivery to target onset) across electrodes; the second one focused on the electrode of interest (FC2, the closest to the stimulated right FEF region) across time and frequencies. Direct planned comparisons (two-tailed paired t-tests at p < 0.01) between active and sham trials separately for both rhythmic and random TMS patterns, and direct comparisons between rhythmic and random trials separately for both active and sham TMS were performed. For the first analysis, topographical maps of power and inter-trial coherence at specific frequency and time window of interest (30 Hz and [−133 0] ms; 0 being the target onset) were compared with paired t-tests calculated for every electrode. Similarly, for the second analysis, power across time and frequencies aligned to the target onset (commonly referred to as event-related spectrum perturbation, ERSP) and inter-trial coherence (ITC) at the electrode FC2 were compared with paired t-tests calculated for every time-frequency point during a restricted time window of interest [−300 200] ms. To correct for multiple direct planned comparisons, electrodes or time-frequency points that reached significance in the paired t-test were clustered and a non-parametric permutation test was applied on these clusters (10000 permutations, alpha = 0.05^38,43^, see Figs 2 and 3 for results, only significant results are displayed). Because EEG data is highly correlated in space and exhibits physiological effects that last over several time points (i.e. an effect is likely to be spread over adjacent sensors and consecutive time points) cluster-based permutations is a highly sensitive method for solving the multiple comparison problem^43^. However, in the case of a factorial design, there is no consensus on how to permute the data to correctly control for multiple comparisons when evaluating interaction effects between multiple factors^44,45^. For this reason, and driven by hypotheses of a different effect of rhythmic and random stimulation patterns on oscillatory activity, we chose to compute direct pairwise comparisons between our conditions.

#### Synchrony entrainment: evoked oscillations

**Figure 4:**
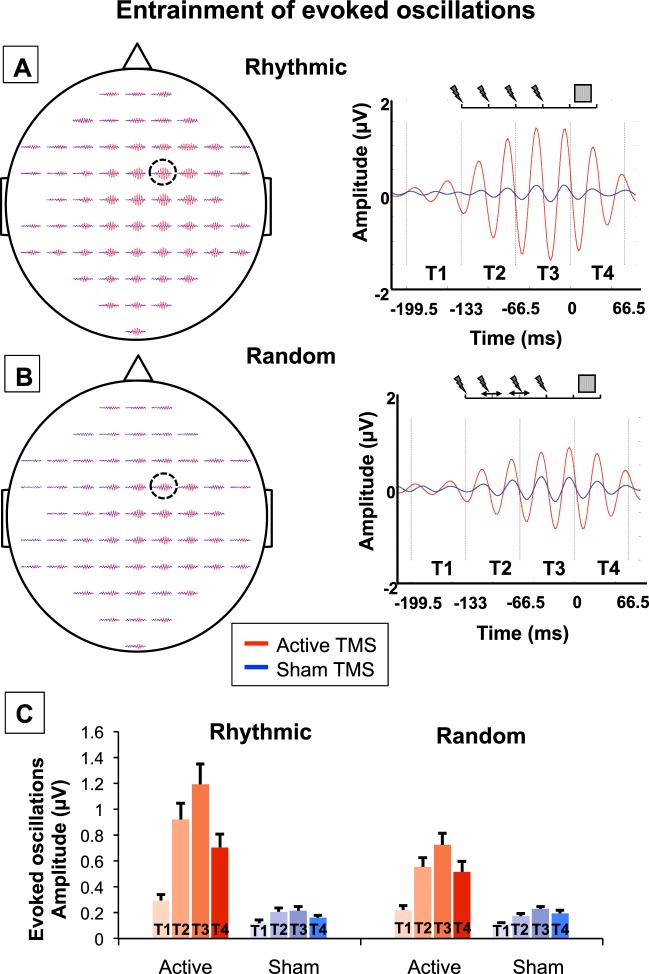
Causal impact of right FEF stimulation on evoked high-beta oscillations. Evoked oscillations (25–35 Hz, [−199.5 66.5] ms, [−2 2] µV) for rhythmic **(A)** and random **(B)** active/sham stimulation patterns for each of the 60 EEG scalp electrodes (left column; the location of electrode FC2, i.e., the closest to the stimulated right FEF, is indicated with an open circle) and at FC2 (right column). Vertical black dotted lines delineate the epochs employed for the analyses (T1: Pre TMS, T2: TMS burst part 1; T3: TMS burst part 2 and T4: Visual Target). Blue and red colors respectively represent the *sham* and *active* TMS conditions. Notice progressive increases in the amplitude of high-beta evoked oscillations (25–35 Hz), reaching higher levels during *rhythmic* than *random* active patterns throughout the course of 4-pulse stimulation patterns followed by a rather abrupt decay after the last pulse of the burst. **(C)** Amplitude (mean and standard error) of evoked oscillations (25–35 Hz) for *rhythmic* and *random* active/sham stimulation patterns across the 4 time-windows of interest (T1: Pre TMS; T2: TMS burst part 1; T3: TMS burst part 2; and T4: Visual Target). Due to the complexity of representation of interaction effects, significant statistical results are not shown in the figure. Notice, however, that active *rhythmic* patterns caused higher amplitude increases of evoked oscillations than active *random* patterns (significant *stimulation pattern x stimulation condition* interaction). In addition, we found a progressive build-up of evoked oscillations along the course of the 4-pulse burst (amplitude T1 < T2 < T3), and a decay following the offset of the stimulation (T4 < T3, significant *stimulation condition* x *time window* interaction).

The ensuing procedure aimed to estimate evoked oscillations, defined as high-beta EEG signals time-locked to the target onset^46^ and consequently to the TMS pulses forming the stimulation patterns (all four pulses of rhythmic TMS patterns, and the first and last pulses of random TMS patterns). This analysis followed a similar rational as the one used to calculate ITC, mentioned just above, employed to estimate phase alignment. Nonetheless, supplementing the latter, it allowed a finer analysis of EEG activity across different time windows before, during, and after the delivery of each TMS burst type. More specifically, it allowed us to make a difference between remaining artifacts and/or a repetition of evoked potentials (resulting in constant evoked amplitudes) and genuine entrained oscillations (yielding increased evoked amplitudes across the burst). Thus, for this analysis, data were filtered ([25 35] Hz, see Fig. 4 for results). Then, four time-windows of interest were defined as follows: T1: Pre TMS [−199.5 −133] ms; T2: TMS burst 1^st^ half (post pulses 1&2 for rhythmic TMS patterns) [−133 −66.5] ms; T3: burst 2^nd^ half (post pulses 3&4 for rhythmic TMS patterns) [−66.5 0] ms; T4: Visual Target [0 66.5] ms. The length of the time-windows was arbitrarily chosen to cover two cycles of a 30-Hz oscillation. The amplitude of evoked oscillations was calculated by averaging the filtered data across trials, before averaging within each time window. The resulting amplitudes were analyzed using a trends-based repeated measures ANOVA with *stimulation pattern* (rhythmic, random), *stimulation condition* (active, sham) and *time window* (both linear and quadratic coefficients) as within-participant factors (see Fig. 4C; note similar standard errors across conditions).

#### Frequencies of interest

In line with earlier findings and the most current mechanistic understanding of stimulation-induced entrainment^15,24^, the entrainment phenomenon occurs mainly in a frequency band centered around the stimulation frequency^21,47^. On that basis, and also building on our prior experience in the domain^10–12,27^, our task design and analytical and statistical strategy were directed to assess changes in oscillatory activity centered in the TMS frequency (30 Hz) delivered to the right FEF. Specifically, we did not quantify higher gamma activity, potentially contaminated by muscles artifacts and line noise. Similarly, modulation of frequencies lower than mid-alpha, which developed at much longer time scale than the duration of the TMS burst and the temporal window of interest chosen for our analyses, was not statistically assessed. For these reasons, we cannot rule out the possibility that 30 Hz FEF TMS also modulated oscillatory activity outside of the high-beta range.

### Behavioral data analyses

Visual detection performance was assessed with perceptual detection sensitivity (d’) and response criterion (β). These two outcome measures are employed in Signal Detection Theory (SDT) to characterize detection performance when it can be strongly influenced by belief (e.g., when stimuli are presented around the perceptual threshold)^48^. Perceptual detection sensitivity is a bias-free measure of the participants’ ability to detect a target, whereas response criterion describes the relative preference (bias) of participants for one response over the alternative one in case of doubt (i.e., in our case, a preference for ‘yes, I saw the target’ over ‘no, I did not see it’).

To compute these measures, trials in which the location of the target was correctly reported by participants were considered correct detections or “hits”; trials in which the presence of the target was not acknowledged were considered “misses”; trials in which participants reported a location for a target that was not present were considered “false alarms”; trials in which the target was absent and participants correctly reported not having seeing it were considered “correct rejections”. Trials in which the location of a present target was incorrectly reported were counted as “errors” and excluded from the main analyses (given the impossibility to distinguish whether participants incorrectly detected the target or correctly detected it but pressed the wrong button). Following an established procedure, zero false alarms were replaced by half false alarms (0.5) in order to calculate d’ and β measures^49^. Perceptual detection sensitivity and response bias were calculated as follows: d’ = ϕ^−1^(H) − ϕ^−1^(FA) and β = N(ϕ^−1^(H))/N(ϕ^−1^(FA)), where ϕ^−1^ is the z-transform, N the un-cumulated density function, H the hit rate, and FA the false alarm rate.

**Figure 5:**
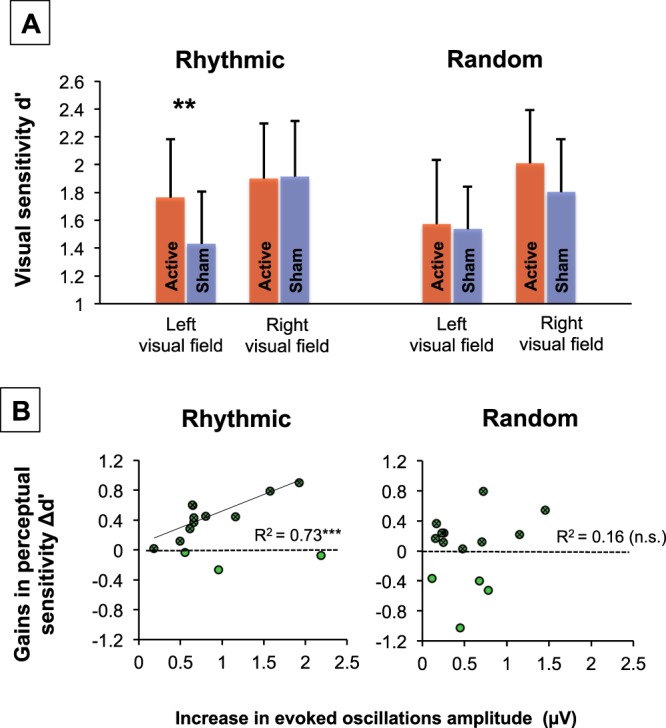
Causal impact of right FEF stimulation on visual detection and relationship with entrained high-beta oscillations. (**A**) Group impact of active/sham rhythmic and random patterns delivered to the right FEF on the detection of near-threshold targets presented in the left or right visual fields (means and standard errors; statistical comparison: **p < 0.01). Importantly *rhythmic* (but not *random)* right FEF active stimulation which, according to EEG evidence (see Figs 2 and 3), increased high-beta power and inter-trial coherence, also increased visual detection sensitivity (d’) for targets displayed in the left visual hemifield. (**B**) Correlation plots between levels of high-beta entrainment (estimated through increases of amplitude of evoked oscillations between active and sham TMS) and visual detection gains (d’ active TMS - d’ sham TMS) with *rhythmic* (left) or *random* (right) active TMS patterns for targets presented in the left visual field. Green dots represent all participants (n = 14). Dark green crossed dots represent pools of participants (n = 11 for high-beta rhythmic TMS, n = 10 for random TMS) who experienced visual detection sensitivity (d’) increases with right FEF stimulation. For high-beta rhythmic TMS, a linear correlation with only the latter selected cohort of participants (black regression line) proved highly significant, whereas for random TMS, no correlation reached significance (***p < 0.001; n.s. non-significant).

For statistical analyses, d’ and β were each subjected to a 2 × 2 × 2 repeated-measure ANOVA with *stimulation pattern* (rhythmic, random), *stimulation condition* (active, sham) and *target location* (left, right) as within-participant factors. Pairwise comparisons between specific conditions were performed with t-student tests. The ANOVA on response criterion (β) did not reveal any significant main effect or interaction (all the p > 0.11). Thus, only effects on perceptual detection sensitivity (d’) are presented in the main text of the manuscript (see also Fig. 5A; note similar standard errors across conditions).

Correlations between significant rhythmic or random TMS-driven (active minus sham) modulation of evoked oscillations (measured for time window T3, given that according to prior literature^21^, we predicted high-beta entrainment to reach its peak towards the end of rhythmic stimulation bursts), and rhythmic or random TMS-driven (active minus sham) modulation of visual detection sensitivity (d’), were tested with a Pearson’s correlation coefficient test.

### Further statistical considerations

Statistical significance was set at *p* < 0.05 and tests were two-tailed. To further strengthen the validity of our findings, we indicated the 95%-Confidence Intervals (CI) and Cohen’s d effect size (with correction for low sample^50^; interpretation: d = 0.2: small; d = 0.5: medium; d = 0.8: large effect size^51^) when appropriate. To further test our main conclusions, we also calculated Bayes factors. For this, we used a uniform interval bound by the compared values (with opposite signs); when necessary, we corrected for the number of degrees of freedom as indicated by Dienes (2014) (interpretation: B < 0.3: substantial evidence for the null hypothesis; B > 3: substantial evidence for the alternative hypothesis). Finally, we performed bootstrap statistics based on 1000 correlation coefficients calculated on datasets drawn from the original dataset with replacement (alpha = 0.05). To compare the strength of different correlations, we calculated unpaired t-tests between the Bootstrap distributions of correlation coefficients and the Z_2_* as indicated in Steiger^52^. Note that all reported analyses were planned *a priori* except when the contrary is explicitly indicated in the text.

## Results

Our group of participants performed a visual detection task, where they had to report if they detected a laterally presented target or not and, if they did, where on the screen it appeared. Within the same blocks of trials, active or sham TMS was delivered to the right FEF shortly before the onset of the visual target (see Fig. 1A,B). Two different TMS patterns were delivered in separate blocks: rhythmic (30 Hz) or random (no specific frequency) bursts made of 4 consecutive TMS pulse for a total duration of 100 ms (see Fig. 1C). EEG was continuously recorded during the task.

### Impact of high-beta right frontal stimulation on event-related spectrum perturbation

Event-related spectrum perturbation (ERSP) analyses of our EEG data assessed modulations of power across different time-frequency bins and electrodes. We first examined the modulation of power for all electrodes at a specific frequency (30 Hz) and time window ([−133 0] ms; 0 being the target onset; see Fig. 2A) of interest. Second, we examined the modulation of power at the electrode of interest, i.e., the closest to the stimulated right FEF (FC2), for a broader frequency range ([6 45] Hz) and time window ([−500 500] ms, see Fig. 2B).

The first analysis revealed that both *rhythmic* and *random* active right FEF stimulation significantly increased high-beta power at 30 Hz, compared to their associated sham control patterns. This increase was rather widespread, reaching significance in scalp electrodes over frontal, central, and parietal sites for the active vs. sham *rhythmic* TMS conditions. In contrast, the comparison between active vs. sham *random* TMS conditions showed a more spatially restricted impact centered over right frontal sites (see Fig. 2A, the bold black dots on the Statistics maps indicate electrodes showing significant differences between active and sham TMS conditions). No consistent differences in 30 Hz power (with the exception of two isolated parietal electrodes) were found when directly comparing active *rhythmic* vs. active *random* stimulation. The second analysis revealed that high-beta power on FC2 recordings was significantly enhanced during *rhythmic* stimulation (active vs. sham comparison) around 30 Hz (~ [25–45] Hz frequency band), and during *random* stimulation (active vs. sham comparison) across the whole spectrum of tested frequencies ([6–45 Hz]) (see Fig. 2B).

These analyses suggest that both rhythmic and random active TMS patterns (which only differed with regards to the temporal distribution of their 4 pulses) significantly enhanced high-beta power. The spatial and frequency signatures of these effects appear potentially distinct; however, such differences did not reach statistical significance when *rhythmic* and *random* TMS were directly compared.

### Impact of rhythmic activity on the phase alignment of right frontal activity

The impact of stimulation on phase alignment was studied through the analysis of inter-trial coherence (ITC), a measure that assesses the consistency across trials of a neurophysiological signal phase. Data from either the complete electrode grid (30 Hz and [−133 0] ms) or specifically from the FC2 electrode (extended time-frequency window) revealed that both *rhythmic* and *random* stimulation significantly increased phase alignment at the high-beta band. Nonetheless, the direct comparisons between the two active conditions (which only differed in the temporal distribution of their 4 TMS pulses) showed higher ITC for the active *rhythmic* than for the active *random* stimulation condition (see Fig. 3).

### Impact of rhythmic TMS activity on frontal evoked oscillations

Analyses assessing the magnitude of time-locked high-beta [25 35] Hz filtered EEG signals (i.e., evoked oscillations) aimed to further prove that active rhythmic 30 Hz patterns entrained local oscillations. To that end, we averaged the amplitude of evoked oscillations within 4 time-windows of interest (T1 to T4, see Fig. 4 for details).

Figure 4 reveals progressive increases of the beta oscillations across the first 3 time windows (T1 < T2 < T3), followed by a decay in the last time window (T3 > T4). Such a quadratic trend appeared larger for active than sham conditions, and more so for rhythmic than random patterns. To evaluate the significance of these observations, a trends-based ANOVA with the *stimulation pattern*, *stimulation condition* and *time window* factors, the latter being evaluated by linear and quadratic coefficients, was used. The ANOVA yielded no evidence of a lack of fit for the model (p = 0.69) and showed significant main effects of *stimulation pattern* (F(1,13) = 15.26, p < 0.001), *stimulation condition* (F(1,13) = 177.42, p < 0.0001) and *time window* (linear: F(1,13) = 23.26, p < 0.0001; quadratic: F(1,13) = 46.10, p < 0.0001). More importantly, this ANOVA also showed a significant *stimulation pattern* (rhythmic, random) x *stimulation condition* (active, sham) interaction (F(1,13) = 15.69, p < 0.001), revealing that increases in the amplitude of evoked oscillations for active vs. sham patterns were significantly higher for *rhythmic* than for *random* stimulation (p < 0.001, CI = [0.14 0.41], d = 0.93, B_U[−0.33 0.60]_ = 111; both active vs. sham differences were highly significant when tested separately, p < 0.0001; for rhythmic: d = 2.14; for random: d = 1.79). Furthermore, *stimulation condition* (active, sham) interacted with both the linear (F(1,13) = 11.57, p < 0.001) and the quadratic coefficients (F(1,13) = 25.77, p < 0.0001) of the time-window; the (positive) linear coefficient was higher for active than for sham stimulation (CI = [0.60 1.31], d = 1.21, B_U[−0.18 1.13]_ = 44), and likewise, the (negative) quadratic coefficient was lower for active than for sham stimulation (CI = [−0.24 −0.10], d = 1.11, B_U[−0.03 0.20]_ = 9745). The *stimulation pattern* (rhythmic, random) interacted with the quadratic coefficient (F(1,13) = 4.90, p < 0.05); the (negative) quadratic coefficient was lower for rhythmic than for random stimulation (CI = [−0.15 −0.01], d = 0.46, B_U[−0.08 0.16]_ = 3.02). Finally, there was a trend towards significance for the 3-way interaction including the quadratic component (F(1,13) = 3.54, p = 0.061), which could be explained by a larger active vs. sham decrease of the (negative) quadratic coefficient for rhythmic than for sham stimulation (p < 0.05; CI = [−0.26 −0.03]; d = 0.76; B_U[−0.10 0.24]_ = 2.26; both active vs. sham differences being significant when tested separately, p < 0.01; for rhythmic: d = 1.34; for random: d = 1.14).

To summarize, evoked high-beta oscillatory activity entrained by rhythmic 30-Hz patterns prior to the onset of the visual target increased progressively across the 4-pulse TMS burst, and decayed rather rapidly after the offset of the stimulation pattern. Together with the increase of power (for both rhythmic and random stimulation) and ITC (higher for rhythmic than for random stimulation), these results support the ability of *rhythmic* 30 Hz patterns to entrain, to a higher extent than *random* patterns, high-beta oscillations in the right FEF. This suggests that rhythmic stimulation is the optimal TMS pattern to enhance power and increase phase alignment, i.e., to entrain *oscillatory* activity at the stimulation input frequency.

### Impact of right frontal rhythmic stimulation on visual detection

The contiguous behavioral consequences of high-beta oscillation entrainment on visual detection (i.e., visual detection sensitivity d’) were explored with a 2 × 2 × 2 repeated-measure ANOVA with the factors: *stimulation pattern* (rhythmic, random), *stimulation condition* (active, sham) and *target location* (left, right). This ANOVA showed main effects of *stimulation condition* (real, sham: F(1,13) = 5.33; p < 0.05) and *target location* (right, left: F(1,13) = 10.14; p < 0.01), supporting higher visual detection under active than sham stimulation and also for targets displayed in the right than the left visual hemifield (Fig. 5A).

A trend towards statistical significance for the triple interaction (F(1,13) = 3.97; p = 0.068) was found. Despite the fact that this interaction was only close to significance, a direct comparison is of high interest for testing our *a priori* hypothesis that rhythmic and random effects on perception might be different^10,12,27^. Thus, we performed t-tests, which showed that rhythmic activity increased visual detection sensitivity (d’) for targets presented in the left, i.e. contralateral to the stimulation (active vs. sham p < 0.01, CI = [0.14 0.53], d = 0.72), but not in the right visual hemifield (active vs. sham p > 0.88, CI = [−0.23 0.20], d = 0.03). In contrast, random stimulation failed to influence visual detection sensitivity for targets in either of the two hemifields (both active vs. sham comparisons p > 0.11 and d = {0.08; 0.47}) (see Fig. 5A). For the left visual field, the Bayes factor fell short of the conventional criteria for substantial evidence of higher increase following rhythmic than random patterns (B_U[−0.04 0.33]_ = 2.62) whereas for right visual field, the evidence was less conclusive (B_U[−0.01 0.21]_ = 1.71).

Our analyses suggest that brief *rhythmic* stimulation patterns entrained high-beta activity in the right FEF prior to the onset of a lateralized visual target, and that such entrained oscillatory activity is causally related to the improvement of visual detection of targets displayed in the left visual hemifield.

### Correlations between entrainment and visual detection gains

Aiming to provide additional support for a causal link between the entrainment of right frontal high-beta oscillations and improvements of visual detection, we correlated the outcome measure gauging levels of entrainment (i.e., active minus sham rhythmic TMS differences in the amplitude of evoked oscillations for time window T3) and gains of visual detection (i.e., active minus sham rhythmic TMS visual detection sensitivity (d’) differences for left hemifield targets).

When all participants were included in the analysis, the Pearson’s correlation coefficient between these two measures failed to reach significance (R^2^ = 0.06; p = 0.39; df = 12; p_bootstrap_ = 0.90). Given this null result, we formulated an additional *a posteriori* hypothesis (i.e., not initially planned or considered), supported by published evidence indicating that inter-individual differences in structural connectivity between the FEF and right posterior parietal regions could explain a failure of TMS to modulate visual detection when targeting this right frontal area^11,12,53^. On that basis, we decided to perform the same analysis including the 11 participants who did show increases of visual detection sensitivity after 30 Hz rhythmic stimulation delivered to the right FEF. It revealed a highly significant correlation between these two variables (R^2^ = 0.72; p < 0.001; df = 9, Fig. 5B left; p < 0.05 after Bonferroni correction; p_bootstrap_ < 0.05). These results show that when rhythmic stimulation enhanced visual detection, the magnitude of the evoked oscillation entrainment correlated significantly with increases in visual performance for left targets.

Several observations strengthen the specificity of this significant correlation between evoked oscillations entrained by rhythmic TMS patterns and behavioral outcomes. First, we failed to find a significant correlation between the same variables for *random* stimulation patterns, neither when considering all participants, nor when selecting the same 11 participants mentioned above or a newly selected cohort of participants attesting increases of visual detection sensitivity for left targets under *random* stimulation (all 3 analyses p > 0.25 and p_bootstrap_ > 0.25). Second, the significant correlation coefficient for active *rhythmic* stimulation with the selection of 11 participants proved significantly higher than the non-significant correlation coefficient shown for active *random* stimulation (p < 0.0001; Z_2_* = 2.27). Third, none of the correlations between increases of evoked oscillations (active minus sham) for time-window T3 and increase of visual detection sensitivity (active minus sham) for right targets (instead of left targets) proved significant (p > 0.05 and p_bootstrap_ > 0.20 for both *rhythmic* and *random* stimulation conditions, regardless of the selection of participants considered). Moreover, the significant correlation coefficient for *rhythmic* pattern and visual detection sensitivity gains for left targets was significantly higher than the non-significant correlation coefficient for right targets (p < 0.0001; Z_2_* = 2.86 for rhythmic patterns and p < 0.0001; Z_2_* = 2.47 for *random* patterns).

## Discussion

The present study replicates previous findings from our group^10–12^, reporting perceptual enhancement with the delivery of TMS bursts over the right FEF (significant main effect of stimulation comparing active vs. sham stimulation). Crucially, our findings also contribute electrophysiological evidence suggesting that TMS entrains high beta oscillations (with both rhythmic and random stimulation, but to a higher extent with the former than the latter). This result informs on the missing causal link between the high-beta oscillatory signature in the right FEF and conscious perception, which is here estimated by measuring visual sensitivity in the context of a lateralized visual detection task for near threshold targets.

Electrophysiological (EEG) recordings revealed that rhythmic stimulation entrained oscillations at the input frequency band (~30 Hz). Indeed, both *rhythmic* and *random* patterns increased high-beta power. Nonetheless, *rhythmic* patterns induced significantly higher levels of phase alignment than *random* activity. Moreover, the amplitude of evoked oscillations, phase-aligned to the stimulation, built up during the course of a 4-pulse stimulation burst, and achieved significantly higher amplitude for *rhythmic* than for *random* stimulation. It then decayed rather rapidly after its offset. These outcomes support the ability of rhythmic TMS to noninvasively manipulate local synchrony in circumscribed cortical regions and impose specific patterns of oscillatory activity, which might serve to explore, enhance or even restore human behaviors in the near future.

Among the two main techniques currently available to manipulate oscillatory activity in humans, namely rhythmic TMS^10–12,14,15,21,22,54–57^ and transcranial alternative current stimulation (tACS)^58–63^, we opted for the former, given its higher focality and special ability to entrain on a trial-by-trial basis episodic events of oscillatory activity during specific time-windows, which is crucial for probing focal contributions to ongoing human cognitive processes and behaviors. Nevertheless, the use of tACS could prove of future interest to induce in a much more flexible manner lasting synchronization over wide cortical regions, with the ultimate aim to improve visual perception in healthy individuals or brain damage patients.

Entrainment of biological rhythms emerges from a progressive phase alignment of different local oscillators following the rhythms dictated by either internal or external “pacemakers”, which in our study were provided by focal rhythmic TMS^15,21,24^. Consequently, simultaneous EEG recordings should show both, increases in power at the input frequency and a progressive phase alignment.

The progressive build-up of high-beta oscillations during the course of the burst featured in our data grants convincing support in favor of rhythmic entrainment. It disentangles the synchronization of neural assemblies at the stimulation frequency from a mere injection of rhythmic activity arising from evoked potentials triggered by each individual TMS pulse^64,65^. Indeed, whereas evoked potentials generated by individual pulses tend to keep a similar amplitude, increases of post-pulse activity throughout the course of the burst (a measure that in our study proved significantly higher during *rhythmic* than *random* active TMS patterns) are most likely associated with a phase alignment of local cortical oscillators.

Taking these criteria into account, our data indicate that while both active *rhythmic* and *random* stimulation patterns yielded power increases within the high-beta band compared to their associated sham bursts (see statistical maps Fig. 2), phase alignment was higher for rhythmic than random stimulation (see statistical maps Fig. 3). Consequently, 30 Hz *rhythmic* patterns led to superior increases in the amplitude of evoked high-beta oscillations and resulted in stronger entrainment of beta rhythmic activity (see Fig. 4). Analogously, active stimulation (taking both rhythmic and random stimulation into consideration) increased visual detection sensitivity (main effect of stimulation factor). Although statistically fragile, two additional results further support an association between oscillatory entrainment and increased visual perception. First, following a trend towards significance (p = 0.068) for a three-way interaction between factors (stimulation condition x stimulation pattern x target location), we reported that rhythmic but not random active TMS patterns increased visual detection sensitivity of targets presented in the contralateral hemifield (see Fig. 5A). Second, only for the 11 participants (out of 14) who displayed enhancements of visual detection sensitivity with rhythmic TMS patterns, entrainment of high-beta oscillations scaled with visual detection increases (see Fig. 5B). It must be noted that the selection of this subgroup of participants was not planned *a priori* but implemented *post-hoc*, only after a lack of significant correlation integrating the whole cohort of participants was verified. Taken together, these results support a causal role for high-beta right frontal rhythms in mediating access to visual consciousness, here estimated by measuring visual detection sensitivity.

Pioneering evidence in the field of rhythmic non-invasive stimulation has suggested that the entrainment of oscillations results from the alignment of local oscillators operating at their so-called “natural” frequency^21^ or in neighboring oscillation bands^54^. Since brain areas tend to naturally oscillate within specific frequency ranges^66^, local synchrony within a given cortical location cannot be easily imposed at any frequency. In our study, the successful entrainment of high-beta oscillations in the human right FEF, a region of the fronto-parietal network that synchronizes at this same frequency range during the allocation of endogenous spatial attention^7,8^, supports this view. Indeed, developing extensive knowledge on the status of local and network physiological brain activity, and most particularly features of local and interregional synchrony recorded via EEG, is part of the recently established *information-based approach* to non-invasive stimulation, aiming to guide the selection of TMS parameters and optimize the use of these tools in exploratory or applied clinical settings^67^.

Prior evidences in favor of high-beta power increases over both right frontal and parietal areas also suggest that oscillation entrainment in the FEF spreads to interconnected areas across a right-lateralized fronto-parietal network^11,12^. Therefore, improvements of visual performance are likely to be mediated by an engagement of top-down attentional orienting processes. Such effects would be subserved by a right dorsal fronto-parietal network^16,25^, synchronizing at a high-beta frequency^7,8^ and related to the demonstrated ability of attentional orienting mechanisms to facilitate the detection of lateralized near-threshold visual targets^68^.

Two-alternative forced choice visual discrimination tasks, in which participants are required to take a guess whenever they believe that they did not see the stimulus (and in which responses are labeled as correct or incorrect) are generally believed to measure objective visual performance. On the contrary, visual detection tasks such as the one used in the present study, in which participants can report not having seen the target (without being incorrect), are believed to measure subjective perception^69,70^. Of interest, although these two types of measure can be in excellent agreement^71^, they have been found to be differentially modulated by attention and subserved by different brain oscillations^70,72^. In the present study, we chose to focus on the effect of TMS on subjective perception, i.e., visual awareness, but this decision does not exclude the possibility that rhythmic stimulation can also increase objective visual performance.

Although top-down attention is a selective mechanism often leading to higher visual awareness^73^, the two processes can be dissociated^74^. Examining the interactions between attention and awareness requires the direct manipulation of attention, e.g., by using spatial cues to orient attention to an area of visual space prior to target onset^18,19^. For methodological reasons (essentially the large number of trials needed per conditions for meaningful TMS-EEG analyses), our behavioral paradigm did not directly manipulate visuospatial attention. Moreover, participants were not requested to answer as fast as possible (preventing the analysis of reaction times, which are often used as a proxy for attentional orienting). Hence, on the basis of our data, we can neither confirm nor exclude that the reported effects on right frontal high-beta oscillations and visual detection were subserved by an attentional mechanism. Investigating the specific role of attention in the causal influence of FEF beta activity on visual consciousness will require a specific design and will have to be addressed in future ad hoc experiments.

We would like to emphasize that the possibility to entrain beta oscillations directly by stimulating the right FEF does not necessarily imply that natural beta oscillations are exclusively of cortical origin. Indeed, thalamo-cortical loops have been shown to play a role in both the regulation of cortical oscillations and attentional and awareness processes^75^. Furthermore, such loops might be regulated by dopamine release in the basal ganglia, which has been in turn associated to beta oscillations (13–30 Hz) in motor networks^76^ and to improvements in subjective and objective visual performance^77^. The extent to which the level of dopamine and/or ongoing beta oscillations is influencing the magnitude of TMS-evoked oscillations and the increase of perceptual detection sensitivity was out of the scope of the current study, but remains an interesting topic of investigation for future studies.

Single-pulse TMS over the right FEF has also been shown to speed-up discrimination and/or increase detection and visual awareness^18–20^. This evidence suggests that the enhancement of visual performance and awareness could also result from TMS-driven increases of background activity, drifting closer to threshold, hence helping weak signals to reach perceptual threshold and become visible^20^. Alternatively, enhancement could have resulted from boosting only specific clusters of neurons according to their level of activity^78^. Such effects could result from local activation within the FEF, or from top-down modulations of occipital brain regions, which enhance the gain of incoming visual signals^20^. To this regard, single TMS pulses delivered to the right FEF have been shown to modulate occipital excitability phosphene threshold^79^. Similarly, short rTMS bursts (at ~10 Hz) to the FEF modulated both visual evoked potentials^80^ and fMRI BOLD responses^81^ in occipital areas. Finally, single TMS pulses delivered to a frontal area close to the FEF have been shown to enhance the power of the so-called “natural” beta band activity characteristic from the stimulated region^66^. Thus, improvements of perception following single-pulse TMS over the FEF could have been also associated to increases of beta oscillations. Notwithstanding, this specific hypothesis would need to be explored and demonstrated.

In conclusion, our work provides evidence in humans that episodic oscillations (high-beta 30 Hz activity), operating focally within a cortical region (the right FEF), causally contributes to a specific cognitive process (access to perceptual consciousness, estimated by measuring visual detection sensitivity). Our results also provide support on our ability to entrain episodes of local synchrony subserving a specific cognitive function, opening new avenues to explore, improve and restore behaviors impacted by dysfunctions of brain synchrony.

## Data Availability

Data are available from the corresponding author upon request.
